# Prominent Changes in Cerebro-Cerebellar Functional Connectivity During Continuous Cognitive Processing

**DOI:** 10.3389/fncel.2018.00331

**Published:** 2018-10-01

**Authors:** Gloria Castellazzi, Stefania D. Bruno, Ahmed T. Toosy, Letizia Casiraghi, Fulvia Palesi, Giovanni Savini, Egidio D’Angelo, Claudia Angela Michela Gandini Wheeler-Kingshott

**Affiliations:** ^1^NMR Research Unit, Department of Neuroinflammation, Queen Square MS Centre, Institute of Neurology, University College London, London, United Kingdom; ^2^Department of Electrical, Computer and Biomedical Engineering, University of Pavia, Pavia, Italy; ^3^Brain Connectivity Center, IRCCS Mondino Foundation, Pavia, Italy; ^4^Blackheath Brain Injury Rehabilitation Centre, London, United Kingdom; ^5^NMR Research Unit, Department of Brain Repair and Rehabilitation, Queen Square MS Centre, Institute of Neurology, University College London, London, United Kingdom; ^6^Department of Brain and Behavioral Sciences, University of Pavia, Pavia, Italy; ^7^Brain MRI 3T Center, Neuroradiology Unit, IRCCS Mondino Foundation, Pavia, Italy; ^8^Department of Physics, University of Milan, Milan, Italy; ^9^Brain MRI 3T Center, IRCCS Mondino Foundation, Pavia, Italy

**Keywords:** cerebellum, resting state fMRI, functional connectivity, resting state networks, cognition

## Abstract

While task-dependent responses of specific brain areas during cognitive tasks are well established, much less is known about the changes occurring in resting state networks (RSNs) in relation to *continuous cognitive processing*. In particular, the functional involvement of cerebro-cerebellar loops connecting the posterior cerebellum to associative cortices, remains unclear. In this study, 22 healthy volunteers underwent a multi-session functional magnetic resonance imaging (fMRI) protocol composed of four consecutive 8-min resting state fMRI (rs-fMRI) scans. After a first control scan, participants listened to a narrated story for the entire duration of the second rs-fMRI scan; two further rs-fMRI scans followed the end of story listening. The story plot was purposely designed to stimulate specific cognitive processes that are known to involve the cerebro-cerebellar loops. Almost all of the identified 15 RSNs showed changes in functional connectivity (FC) during and for several minutes after the story. The FC changes mainly occurred in the frontal and prefrontal cortices and in the posterior cerebellum, especially in Crus I-II and lobule VI. The FC changes occurred in cerebellar clusters belonging to different RSNs, including the cerebellar network (CBLN), sensory networks (lateral visual network, LVN; medial visual network, MVN) and cognitive networks (default mode network, DMN; executive control network, ECN; right and left ventral attention networks, RVAN and LVAN; salience network, SN; language network, LN; and working memory network, WMN). Interestingly, a k-means analysis of FC changes revealed clustering of FCN, ECN, and WMN, which are all involved in working memory functions, CBLN, DMN, and SN, which play a key-role in attention switching, and RSNs involved in visual imagery. These results show that the cerebellum is deeply entrained in well-structured network clusters, which reflect multiple aspects of cognitive processing, during and beyond the conclusion of auditory stimulation.

## Introduction

The brain is known to operate through multiple loops forming networks consisting of spatially distributed, but functionally connected regions that continuously share information with each other (van den Heuvel and Hulshoff Pol, 2010). Functional connectivity (FC) can be defined as the temporal correlation of neural activity patterns between anatomically separated brain regions (Friston et al., 1993; Biswal et al., 1997; Friston, 2011). Even at rest, the brain is organized in networks, known as resting state networks (RSNs), in which distinct brain areas exhibit consistent synchronization (Beckmann et al., 2005; Buzsaki, 2006; Sadaghiani et al., 2010; Boubela et al., 2013; Lee et al., 2013; Chen and Glover, 2015). The organization and integration of processing into RSNs can be effectively investigated using independent component analysis (ICA) of $T_{2}^{*}$-weighted fMRI time series as proposed for resting state fMRI (rs-fMRI) (Fox and Raichle, 2007). Traditionally, rs-fMRI is based on the assumption of temporal stationarity, in which linear correlation of BOLD signals has been used to assess FC across regions computed over the whole duration of a single-session scan (Fox and Raichle, 2007). However, there is growing evidence from MRI and animal electrophysiology that RSNs are affected by interfering physiological factors, like arousal and attention, or by performing naturalistic tasks, like watching a movie (Hasson et al., 2010; Kauppi et al., 2010), or listening to a narration (Hasson et al., 2009), so that their stationarity can be lost over time; in this situation, RSN changes can be used to investigate brain dynamics (Deco et al., 2011; Menon, 2012; Elton and Gao, 2015; Hansen et al., 2015; Spadone et al., 2015; Yuste and Fairhall, 2015). Some studies have shown that brain state-dependent FC changes may be related to a variety of different causes such as mental tasks, sleep, and learning (Hutchison et al., 2013). Despite this, how RSNs are recruited and operate during *continuous cognitive processing* under *naturalistic stimulation* in humans *in vivo* is still elusive and it is unclear whether and how long for the RSNs engagement persists after the conclusion of sensory stimulation (Hasson et al., 2009; Berns et al., 2013; Mackey et al., 2013).

Among subcortical structures, special interest has recently been raised by the cerebellum, since growing evidence indicates that, in humans, it plays a relevant role in high-level cognitive and behavioral processing (Schmahmann and Caplan, 2006; Ito, 2008; Buckner, 2013; D‘Angelo and Casali, 2013; Sokolov, 2018). Neuronal recordings in monkeys have demonstrated that the cerebellum does perform sophisticated internal computations essential for motor learning through prediction (Brooks et al., 2015). Moreover, the cerebellum has been shown to connect tightly to associative cerebro-cortical areas and to take part in networks involved in cognitive processing (Castellazzi et al., 2014; Palesi et al., 2015, 2017; Pezoulas et al., 2017) including attention switching, language, imagery and visuo-spatial processing, decision making and reasoning (Ito, 2008; Stoodley, 2012) as well as cognitive control (O’Connell and Basak, 2018). This complex set of operations is expected to involve multiple sub-networks and different cerebellar modules associated with various cortical regions.

In this work, we have developed a framework to estimate and classify FC changes of the RSNs during a multi-session protocol, designed to identify how RSNs FC changes before, during and after listening to a narrated *story*. The story was enriched with elements of movement and mental manipulation, error/novelty detection, working memory and planning to enhance the engagement of working memory and executive functions supported by the cerebro-cerebellar loops.

## Materials and Methods

### Subjects

Magnetic resonance imaging acquisitions were performed on 22 healthy subjects (mean age 27.68 ± 3.53, 11 males, all Italians). All subjects had normal hearing and high educational level (mean years of education = 18.45 ± 1.89). The study was approved by the ethics committee of the IRCCS Mondino Foundation and all subjects provided written informed consent.

### MRI Acquisitions

All subjects underwent MRI examination using a 3T Siemens Skyra scanner (Siemens, Erlangen, Germany) with a 32-channel head coil. For each subject, an fMRI scan consisted in acquiring images using a gradient echo - echo planar imaging (GE-EPI) sequence with TR/TE = 3010/20 ms, flip angle = 89°, voxel size = 2.5 mm isotropic, FOV = 224 mm^2^, 60 slices, 160 volumes, total acquisition time 8.05 min. For anatomical reference a high-resolution 3D T1-weighted (3DT1) volume was collected using a MPRAGE sequence with TR/TE = 2300/2.95 ms, TI = 900 ms, flip angle = 9°, voxel size = 1 mm × 1 mm × 1.2 mm, FOV = 270 mm, 144 sagittal slices, acquisition time 6.31 min. The overall acquisition protocol involved repeating the fMRI scan four times and the total acquisition time was approximately 40 min.

### fMRI Experimental Design

The experiment was designed to study RSN changes in response to an evolving complex cognitive stimulation, resembling the ecological context of every-day life. This was achieved by delivering a narrated story (in Italian). The full text of the story is available as **Supplementary Material** in the original Italian language and in its English translated version. In order to investigate the intervention of the cerebellum in cognition, the story was purposely written following a categorization of functions based on extensive literature revisitation at the physiological, psychological, and neurological level (D‘Angelo and Casali, 2013). Elements that were embedded in the story are *movement and mental manipulation* of objects in space, *error/novelty detection, working memory* and mental *planning* of the consequences of events (prediction). In order to place the story in a context familiar to most listeners, and easy to engage with, the story plot was set in a “school of magic,” which is also the title. The story was recorded on an audio-CD by a female voice. The audio-stimulus had a length of 8.05 min and was presented to the subjects binaurally using a digital audio system.

**FIGURE 1:**
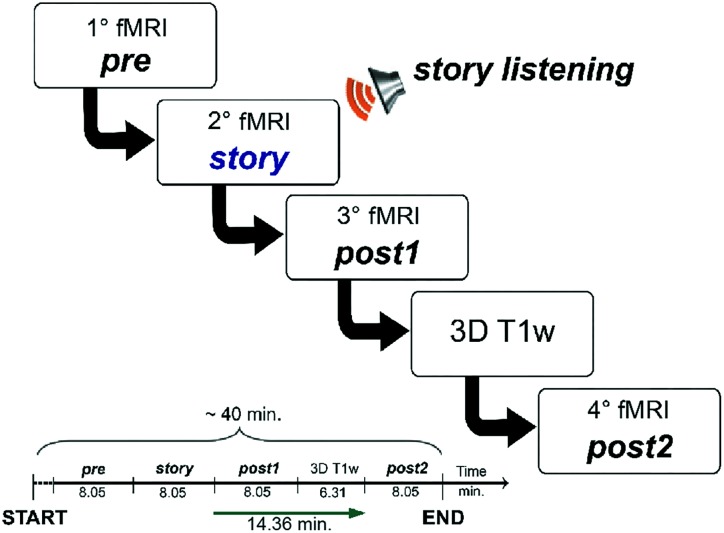
Schematic illustration of the fMRI study design. The acquisition protocol included four consecutive fMRI scans (labeled, respectively, *pre, story, post1*, and *post2*). During the second scan (*story*) subjects were asked to listen to an 8.05 min-long narrated story. The MRI acquisition session had a total duration of 40 min. We imposed an interval of about 15 min between the end of the fMRI with the story (*story*) and the beginning of the last fMRI (*post2*) in order to have a temporal window suitable to detect the evolution of the FC magnitude and spatial extent of each RSN.

The overall acquisition protocol consisted in four repetitions of the rs-fMRI scan, labeled, respectively: *pre, story, post1* and *post2*. The protocol started with the acquisition of the first rs-fMRI repetition (i.e., *pre*), which represented the baseline, i.e., the first control scan of the experiment. During the second rs-fMRI repetition (i.e., *story*), the recording of “School of magic” was played. A third rs-fMRI repetition (i.e., *post1*) was acquired with no stimulus straight after the *story* scan to capture the initial return to baseline. The *post1* acquisition represented the second control scan of the experiment. Since we hypothesized that cognitive processing and working memory-related changes in FC might continue for several minutes (Barnes et al., 2009; Hasson et al., 2009; Gordon et al., 2014), we acquired a second post-stimulus rs-fMRI repetition (i.e., *post2*) as far away as possible in time from the *story* to verify the complete recovery of the resting state baseline status. The *post2* acquisition represented, therefore, the third control scan of the study. In order to keep the overall experiment time feasible and avoid dead periods while the subjects were in the scanner, we performed a high resolution 3DT1 acquisition in between *post1* and *post2*. The time between the end of *story* and the beginning of *post2* was therefore maximized to 14.36 min. The experimental design is summarized in **Figure 1**, where timings between the beginning of each fMRI acquisition and the following one are also indicated.

The participants remained inside the MRI scanner for the entire duration of the multi-session acquisition protocol, which was run without any break. The acquisition protocol was explained to each participant rigorously before the start of the MRI examination, asking them to pay attention to the story narration, which they heard in the scanner for the first time. Participants were also informed that they would be required to fill in a verification questionnaire at the end of the experiment in order to check their level of attention as well as their level of comprehension of the story content. No vocal inputs were delivered to the participants in the scanner after the starting of the MRI acquisition to avoid any sort of listening inputs, other than the naturalistic stimulus, that might interfere with the purpose of the study.

### fMRI Analysis

In this study, rs-fMRI images were treated with the ICA followed by a dual regression technique to identify the RSNs and their changes in relation to the naturalistic stimulation. To investigate the networks’ trends of changes across the four scans of the rs-fMRI protocol, we introduced a relative total change (*rc_T_*) index, derived from specific dual regression maps; we then performed a k-means clustering to group the networks depending on their specific patterns of *rc_T_* changes. We used the k-means results to speculate about the similarity of the RSNs’ responses to the naturalistic task. The details of each analysis are reported below.

#### Data Pre-processing

All fMRI analysis was performed with FSL (FMRIB Software Library, version 5.0.9^1^). Individual subject’s pre-processing consisted in motion correction, brain extraction, spatial smoothing using a Gaussian kernel of full-width-at-half-maximum (FWHM) of 5 mm, and high pass temporal filtering equivalent to 120 s (0.008 Hz). Individual fMRI volumes were registered to the corresponding structural 3DT1 scan using FMRIB’s Linear Image Registration Tool (FLIRT) and subsequently to standard space (MNI152) using FMRIB’s Non-linear Image Registration Tool (FNIRT) with default options.

For each subject, rs-fMRI images were analyzed using the ICA first during pre-processing at single-subject level (single-ICA) for denoizing, using the ICA-based X-noiseifier (FIX) tool (Salimi-Khorshidi et al., 2014) as implemented in FSL. ICA was then applied at group-level (group-ICA) on the pre-processed rs-fMRI data using the Multivariate Exploratory Linear Optimized Decomposition into Independent Components (MELODIC) method in order to characterize the RSNs (Beckmann et al., 2005).

#### Identification of RSNs

For each recruited subject, pre-processed fMRI images underwent group-ICA analysis to characterize RSNs. Specifically, pre-processed functional data, containing 160 time points (volumes) for each subject, were temporally concatenated across subjects to create a single 4-dimensional data set. The dataset was decomposed into independent components (ICs), with an automatic estimation of the number of components, which resulted in spatial maps used subsequently for assessing parameters’ time course over the four fMRI scans. Model order was estimated using the Laplace approximation to the Bayesian evidence for a probabilistic principal component model. Some of the ICs were identified as noise while others as RSNs, based on their frequency spectra and spatial patterns (Beckmann et al., 2005; Smith et al., 2009). In other words, this processing is run on the entire dataset (i.e., the total 4 fMRI acquisitions × 26 subjects = 104 fMRI scans) and decomposes data into spatial maps that are the independent components (ICs) relative to the total processed dataset, or the multi-subject ICA components. This means that ICs are the same for each subject and represent the maps within which inference between scans (*pre, story, post1*, and *post2*) is then evaluated applying the dual regression processing step (see the section “Dual Regression Analysis”).

#### Dual Regression Analysis

A non-parametric permutation test, referred to as “dual regression” technique, was then applied to detect statistically significant differences in the ICs, including the RSNs, among the 4 consecutive repetitions of the fMRI protocol. The dual regression analysis was carried out on the total ICs using age, gender and score of questionnaire as additional covariates (Filippini et al., 2009). In detail, the spatial ICs were used in a linear model fit against each individual fMRI data set (spatial regression), to create matrices that described the temporal dynamics for each component and subject’s session (i.e., *pre, story, post1*, and *post2* for each single subject) separately. Subsequently these matrices were used in another linear model fit against the associated subject’s session data set (temporal regression) to estimate subject’s session-specific spatial correlation map. Spatial maps of all subjects’ sessions were then collected into single dimensional files for each original IC and tested voxel-wise for *group-comparison* contrasts, where in this study *group* means scan session; we also assessed the *group mean effect* (ME) contrasts (labeled *ME_pre_, ME_story_, ME_post1_* and *ME_post2_*) running non-parametric permutation tests (i.e., FSL *randomize* algorithm) (Winkler et al., 2014) with 5,000 permutations. In detail, the *group-comparison contrasts* were first used to identify significant FC changes within the RSNs when looking at the *story* scan vs. all the other scans (*pre, post1*, and *post2*) taken together. This first comparisons (e.g., *story* scan > *all* other scans and *story* scan < *all* other scans) allowed us to investigate whether there were FC differences between the resting state signal in the presence of the story stimulus and all conditions without the stimulus. Direct comparisons of a single scan session vs. another one (e.g., *story* > *pre*) were then assessed for a more detailed analysis of the FC changes within the RSNs during different stages of the experiment. The *group* ME contrasts were instead calculated and fed into a clustering analysis (see the section “Clustering of RSNs Changes” for full details).

For each tested contrast, the resulting statistical maps were corrected for both family-wise error (FWE) and threshold-free cluster enhancement (TFCE). The FWE-TFCE-corrected maps’ voxels that survived a statistical threshold of *p* ≤ 0.05 were considered significant.

#### Clustering of RSNs Changes

For an objective assessment of the RSN changes across the four scans of the fMRI protocol, we performed a clustering analysis using indexes derived from the *group* ME maps we obtained from the *randomize* step (see the section “Dual Regression Analysis”).

For each RSN and for each scan, we used the corresponding ME map to evaluate its spatial extent (SpE, i.e., number of significant non-zero voxels). We used the individual subject’s mean FC maps to obtain values of FC magnitude for each voxel and for each RSN, averaged across the group (i.e., mean FC value of non-zero voxels per scan across subjects) over the areas identified by the corresponding ME maps. For each network, we calculated two parameters that we labeled “relative change in FC” (*rc_FC_*) and “relative change in spatial extent” (*rc_SpE_*) by applying a normalization of FC and SpE specific to each RSN. In detail, the values of FC magnitude and SpE for each RSN and for each scan were normalized, respectively, to the values of FC magnitude and SpE measured in *pre*, as well as at their peak values, as described in formulas (1) and (2):

(1) $${\left[rc_{FC}(j)=\frac{\left(\frac{meanFC_{j}-meanFC_{pre}}{meanFC_{pre}}\right)}{meanFC_{peak}}\right]}_{RSN}$$

(2) $${\left[rc_{SpE}(j)=\frac{\left(\frac{SpE_{j}-SpE_{pre}}{SpE_{pre}}\right)}{SpE_{peak}}\right]}_{RSN}$$

where *j* represents the scans of interest for the evaluation of the *rc* indices (i.e., *pre, story, post1*, or *post2*). We visually inspected the overall RSNs behavioral changes by plotting *rc_FC_* and *rc_SpE_* for each fMRI run. Values of *rc_FC_* and *rc_SpE_* calculated from different fMRI scans were also statistically compared using the repeated measures ANOVA test with Bonferroni correction using SPSS (version 23.0, Chicago, IL, United States).

To have an overall view of the dynamical changes for each RSN between scans over time, we calculated a composite “relative total change” parameter, *rc_T_*, as:

(3) $${\left[rc_{T}(j)=rc_{FC}(j)\cdot rc_{SpE}(j)\right]}_{RSN}$$

Finally, we run a k-means clustering analysis (Duda et al., 2000) using as input argument *rc_T_* values for each RSN and each scan of interest, i.e., *story, post1* and *post2*. The k-means algorithm was run initialized the cluster centroids applying the k-means++ method (Arthur and Vassilvitskii, 2007) and used Euclidean distance as dissimilarity metrics to iteratively (300 repetitions) assign inputs to the closest centroid (Duda et al., 2000). The Silhouette score was used to automatically determine the optimal number of clusters (k) in the data. Specifically, this method computes the average silhouette of observations for different values of k measures, with 2 ≤ k ≤ 10, determining at each run how well each object laid within its cluster. The optimal number of clusters k is the one that maximizes the average silhouette over the range of possible values for k (Kaufman and Rousseeuw, 1990).

The clustering analysis was carried out using the Orange software tool (version 3.9).

## Results

In this study, 22 healthy subjects underwent rs-fMRI, during which they listened to a narrated story. At the end of the recording, the subjects were interrogated about the story content demonstrating their attentive engagement.

### RSN Identification

The results of ICA processing on all fMRI scans resulted in 57 independent components, 15 of which were recognized as plausible networks based on their frequency spectra and spatial pattern (Smith et al., 2009; Castellazzi et al., 2014). The remaining 42 components, reflected artifacts like movement, physiological noise or cerebro-spinal fluid (CSF) partial volume effects.

**FIGURE 2:**
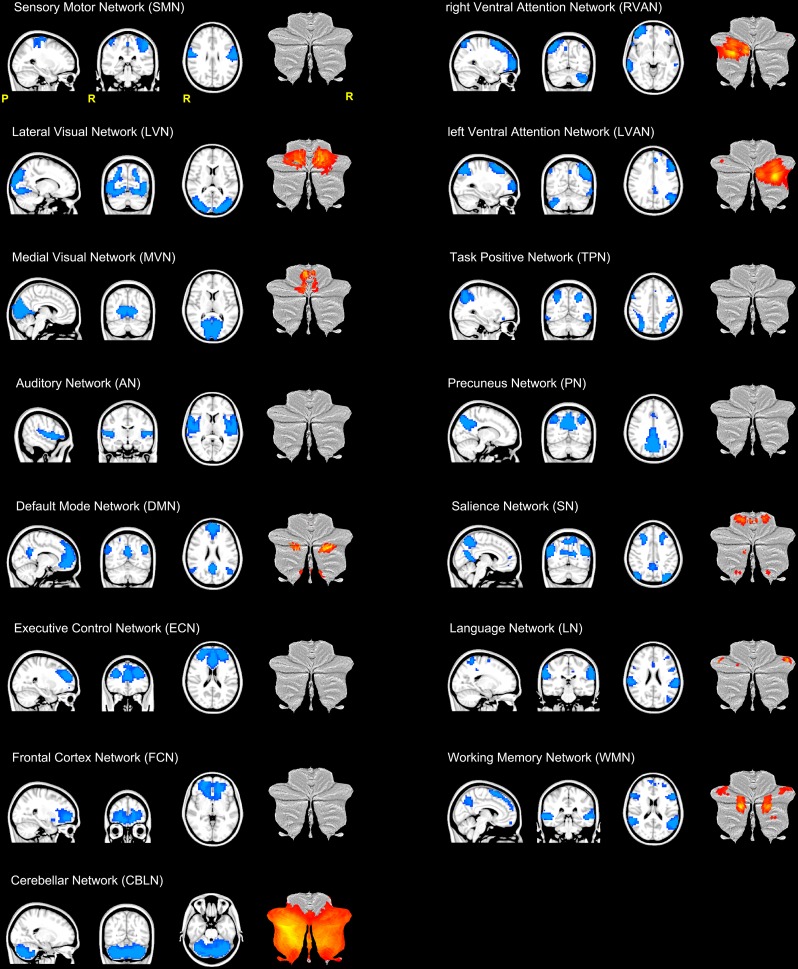
Illustration of the 15 RSNs identified in this study. From top left: sensory motor network (*SMN*), lateral visual network (*LVN*), medial visual network (*MVN*), auditory network (*AN*), default mode network (*DMN*), executive control network (ECN), frontal cortex network (*FCN*), right (R) and left (L) ventral attention networks (*VAN*), task positive network (*TPN*), precuneus network (*PN*), salience network (*SN*), language network (*LN*), working memory network (WMN) and the cerebellar network (*CBLN*). Each RSN is presented as a blue mask on a sagittal, coronal and axial view. For each RSN, the last column of each trio of views show the cerebellar areas (in red-yellow scale) of the network plotted on a flatmap of the cerebellar cortex. With the exception of CBLN which involved the almost the entire cerebellum, nine RSNs showed at least one cerebellar node.

The resulting 15 RSNs were (**Figure 2**): sensory motor network (*SMN*), lateral visual network (*LVN*), medial visual network (*MVN*), auditory network (*AN*), default mode network (*DMN*), executive control network (ECN), frontal cortex network (*FCN*), right (R) and left (L) ventral attention networks (*VAN*), task positive network (*TPN*), precuneus network (*PN*), salience network (*SN*), language network (*LN*), working memory network (WMN), and the cerebellar network (*CBLN*).

SMN, LVN, MVN, and AN are directly implicated in sensory processing, while DMN, FCN, ECN, VAN (RVAN and LVAN), TPN, PN, SN, LN, and WMN are associated with higher cognitive functions (Papanicolaou, 2017). As such, we will refer to this latter group of RSNs as “cognitive” RSNs. The CBLN is usually considered a sensorimotor network, although recent studies highlight its involvement in cognitive circuitries too (Stoodley, 2012).

Furthermore, with the exception of CBLN, which involved almost the entire cerebellar cortex, 9 out of the 15 identified RSNs included clusters in the cerebellum (**Figure 2**). Hence cerebellar nodes were present in the majority of the sensory processing networks (LVN and MVN) and of the cognitive ones (DMN, ECN, RVAN, LVAN, SN, LN, and WMN).

**FIGURE 3:**
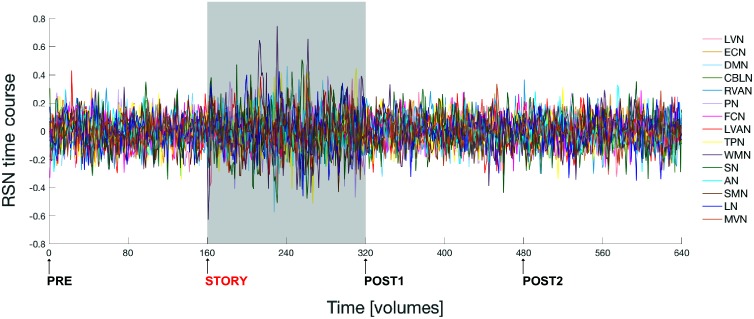
Fluctuations of the ICA time courses associated to the 15 identified RSNs during the entire experiment. The time interval during which the naturalistic stimulus has been delivered (i.e., during *story* scan) is highlighted with a gray panel in the plot. Note that switching brain activity from a *resting state* (i.e., brain condition during the *pre* scan) into an *active state* (condition during the *story* scan) and then back again to *resting state* (*post1* scan condition), over a time scale of several minutes, is associated to a marked change in the amplitude of the RSNs’ time course signals.

**Figure 3** shows the time course signal for each RSN as output by ICA.

### Changes in RSNs During Naturalistic Stimulation

#### Group-Comparisons: FC Changes in RSNs

**FIGURE 4:**
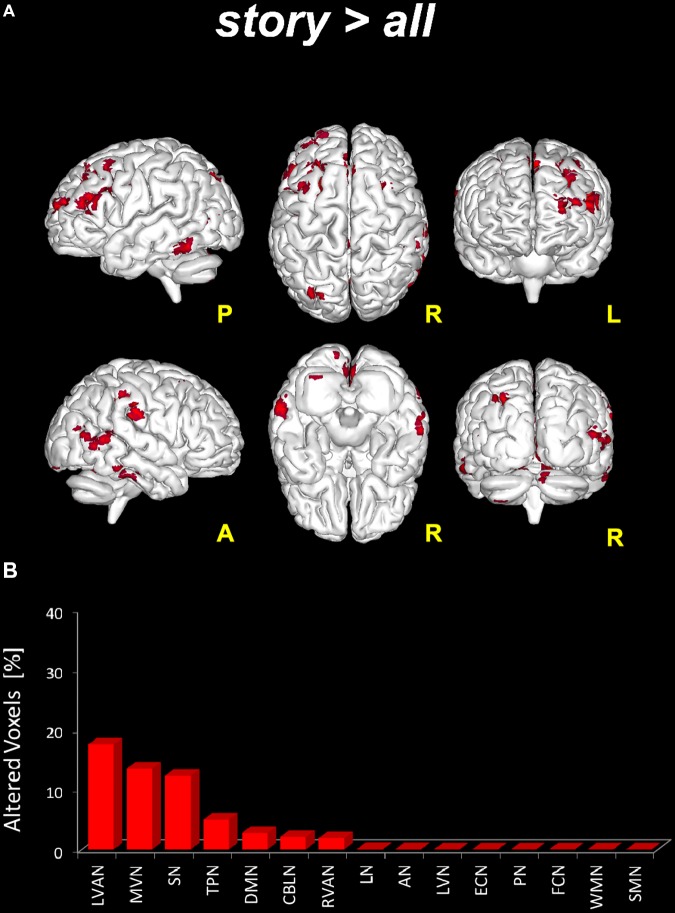
Global FC changes within the RSNs in story > all. On top **(A)**: in red, brain areas showing significantly increased FC (*p* ≤ 0.05, FWE-TFCE-corrected) within the RSNs when comparing the *story* scan to the remaining scans (*pre, post1*, and *post2*) considered altogether as a unique (“all”), i.e., *story* > *all*. The red bar plot on the bottom **(B)** shows, for *story* > *all*, the ranking of the RSNs according to the percentage of their alteration calculated as the per cent ratio between the number of altered voxels in the specific RSN (N_*tstat*_) and the number of voxels of the RSN mask (N_*RNS*_) as it output from ICA.

For each RNS, dual regression analysis was firsts used to reveal the presence of significant FC differences between the *story* scan versus all the remaining scans (*pre, post1*, and *post2*) considered altogether as a single (“*all*”) group (i.e., *story* < *all, story* > *all*). This comparison yielded the following results:

- *story* < *all*: no significant changes were observed when looking for reduced FC areas in *story* versus the remaining scans of the fMRI protocol (i.e., *all* group).
- *story* > *all*: significant areas (*p* < 0.05, FWE-TFCE-corrected) of increased FC in *story* compared to the other scans altogether were found in nine RSNs: MVN, AN, DMN, RVAN, LVAN, TPN, SN, LN, and CBLN (**Figure 4**). Large clusters (>50 voxels) of increased FC were located in the precuneus (involving the posterior DMN and MVN) and in the middle and superior frontal gyrus (LVAN, SN, TPN and the anterior part of DMN). Large clusters (30 < voxels < 50) of increased FC were found in lobule VI of the posterior cerebellum (CBLN, LVAN, and RVAN), *culmen* and lobule V of the anterior cerebellum (CBLN), posterior cingulum (LVAN, SN), thalamus (LN) and superior and middle temporal gyrus (LVAN, TPN, RVAN, and AN).

**FIGURE 5:**
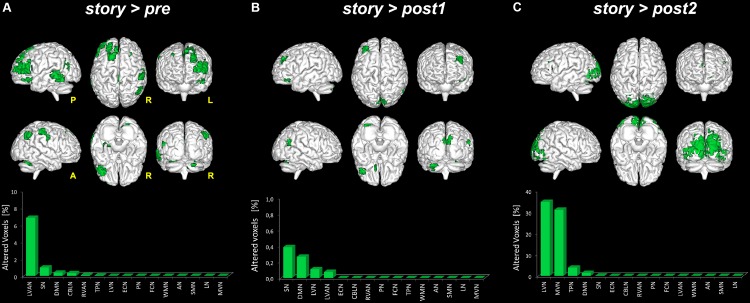
Global FC changes within the RSNs in story > pre, story > post1 and story > post2. The picture show in green the brain areas significantly increased in story > pre **(A)**, *story* > *post1* **(B)**, and *story* > *post2* **(C)**. Below each 3D brain pictures are reported the bar plot which show, for each contrast **(A–C)** the ranking of the RSNs according to the percentage of their alteration calculated as described in **Figure 3** legend.

Given the significant changes observed when considering *story* > *all*, three further comparisons were tested in order to detect significant areas (*p* < 0.05, FWE-TFCE-corrected) of increased FC in *story* compared to either *pre* (i.e., *story* > *pre*), *post1* (i.e., *story* > *post1*) or *post2* (i.e., *story* > *post2*) scans. The following results were found (see also **Figure 5**):

- *story* > *pre*: significant areas of increased FC in *story* compared to the *pre* scan were found in six networks: DMN, RVAN, LVAN, TPN, SN, and CBLN. In detail, large clusters (>70 voxels) of increased FC were found in the cingulate gyrus [Brodmann area (BA)23 and BA3], involving LVAN and DMN, in the superior and middle frontal gyri (BA9-10, BA46) involving LVAN, SN, and RVAN and in the middle temporal gyrus (BA39) involving SN and RVAN. Smaller clusters (20 < voxels < 50) of increased FC were detected in the precentral gyrus (BA4) of the anterior DMN, in the parahippocampal gyrus involving SN, in the inferior parietal cortex (BA40) of LVAN and TPN, and in the cerebellar Crus II of the CBLN and LVAN networks.
- *story > post1*: compared to *post1*, in *story* significant areas of increased FC were found in four networks: LVN, DMN, LVAN, and SN. A large cluster (>50 voxels) of increased FC was centered in the lingual gyrus involving SN. Smaller clusters (20 < voxels < 50) were found in the middle frontal gyrus (BA9-10) involving DMN and SN, in the anterior cingulate gyrus of DMN, in the cuneus (BA19) involving SN and LVN, in the superior temporal gyrus (BA41-42) of SN and in the cerebellar areas of Crus I and lobule VI involving DMN and LVAN.
- *story > post2*: significant clusters of increased FC in *story* compared to the *post2* scan were found in five networks: LVN, MVN, DMN, TPN, and SN. A large cluster (>1,000 voxels) of increased FC was located in the occipital lobe, mainly involving LVN and MVN and extended to the superior parietal lobule, cuneus and precuneus involving areas of DMN, TPN, and SN. Smaller clusters (50 < voxels < 150) were found in the middle (BA9-10) and inferior (BA45-46) frontal gyri involving TPN and DMN. Furthermore, a small cluster (50 < voxels < 100) of increased FC was also detected in the cerebellar Crus I and lobule VI involving LVN.

### Clustering of RSNs According to Their Dynamic Changes

**FIGURE 6:**
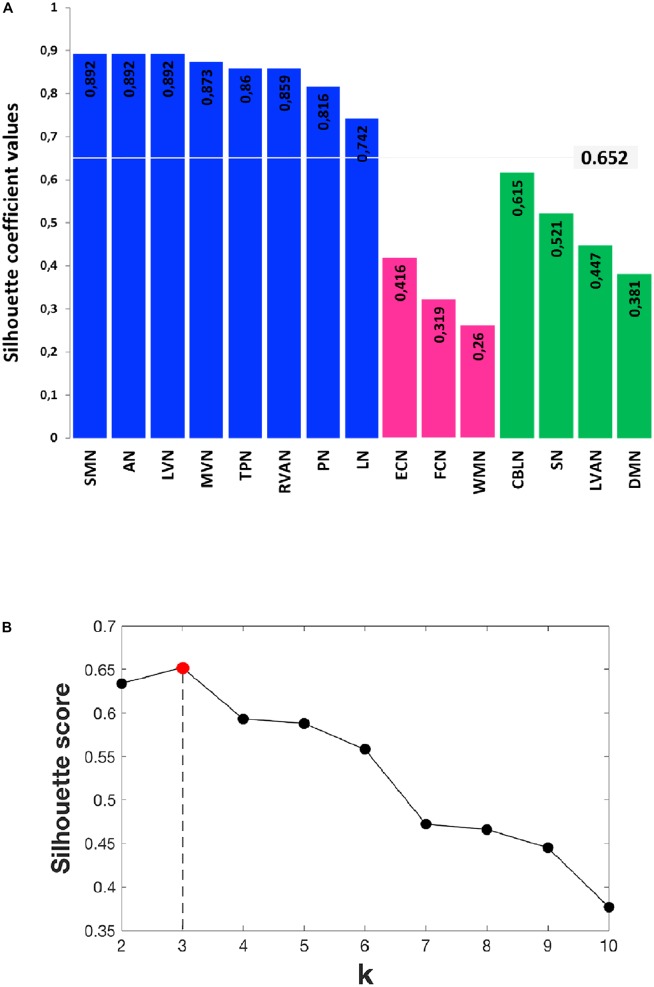
Details of the Silhouette scoring. **(A)** Silhouette plot offering a graphical representation of consistency within the three clusters (C1 in blue, C2 in green and C3 in magenta) identified by k-means with *k* = 3. The Silhouette coefficient values, reported on each bar of the plot, represent a measure of how similar each data instance (i.e., each RSN) is to its own cluster in comparison to other clusters. Specifically, a silhouette coefficient close to 1 indicates that the RSN is close to the center of the cluster, while RNSs with silhouette coefficients close to 0 are on the border between two neighboring clusters. The average value of the Silhouette coefficients reported on the bars is 0.652 and represents the final Silhouette score for *k* = 3. **(B)** Plot showing the average Silhouette scores for different values of k (i.e., different number of k clusters), with 2 ≤ k ≤ 10. Note that, for our data, the maximum Silhouette score (0.652), which corresponds to the optimal number of k clusters to be used for the analysis, is obtained for *k* = 3 (Silhouette score = 0.652).

**FIGURE 7:**
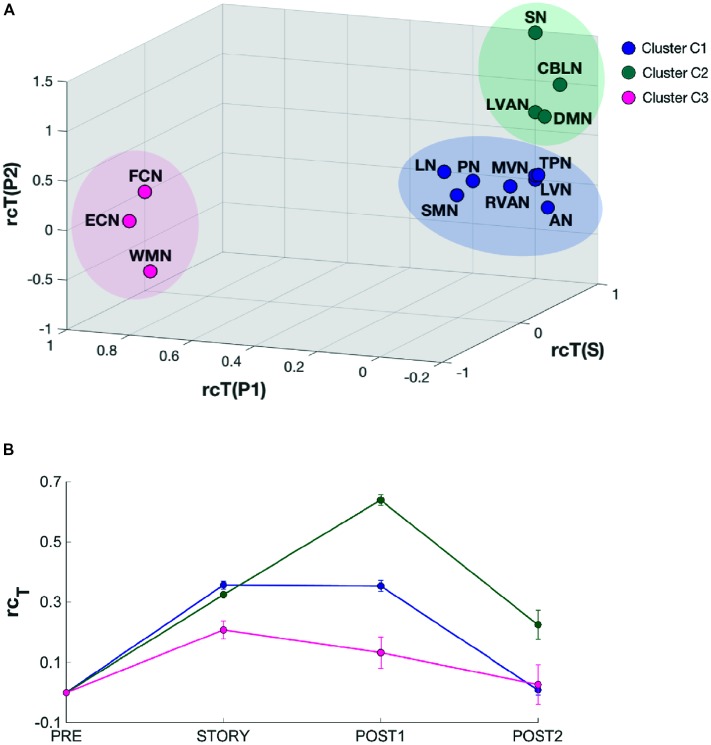
Results of the k-means clustering results analysis for *k* = 3. **(A)** 3D scatter plot showing the position of the elements of each identified cluster (C1, C2, and C3). C1 (blue) contains eight RSNs: LVN, MVN, SMN, AN, RVAN, TPN, PN, and LN. C2 (green) contains four RSNs: DMN, SN, CBLN, and LVAN. The third cluster, C3 (magenta) groups three RSNs: WMN, FCN, and ECN. The axes of the 3D plot represent the three dimensions of the k-means input data: *rc_Tstory_, rc_Tpost1,_ rc_Tpost2_*. **(B)** Plot of the behavioral trends associated to the three clusters. Each trend has been obtained by averaging the *rc_T_* values across scans of the RNSs belonging to the specific cluster. For each trend, the relative standard error has also been reported. C2 cluster has been associated to a “long-lasting” pattern of changes since the mean *rc_T_* trend peaks in *post1* after the listening task which occurs during *story*. On the contrary, C3 has been associated to a “short lasting” *rc_T_* trend which peaks in *story* and recovers rapidly during *post1*, while C1 presents a mixed-trend between those of C2 and C3.

In order to identify possible RSN clusters, a k-means analysis was performed. The Silhouette index resulted maximum for *k* = 3, which indicates the optimal number of clusters to be used for k-means (**Figures 6A,B**). When the k-means algorithm was instructed to group data into three clusters (*k* = 3), the RSNs turned out to be sorted as follows. The first cluster (C1) included eight networks: LVN, MVN, SMN, AN, RVAN, TPN, PN, and LN. The second cluster (C2) included the four RSNs: DMN, SN, LVAN, and CBLN. The third cluster (C3) included the remaining three networks: ECN, FCN, and WMN (**Figure 7A**). These three clusters were functionally related to sensory processing, cognitive processing and working memory (see “Discussion” section below).

In order to quantitatively inspect the kinetics of FC alterations typical of the three clusters identified by k-means analysis, the mean *rc_T_* signal was computed by averaging the *rc_T_* values across scans in each cluster (**Figure 7B**). In C1, the mean *rc_T_* trend peaked in *story* and remained almost at the same level even in *post1* before recovering toward the initial value (as in *pre*) in *post2*. In C2, the mean *rc_T_* trend peaked in *post1* and partially recovered its initial value in *post2*. We called the C2 trend as “long-lasting” pattern of changes. In C3, the mean *rc_T_* trend reached the maximal alteration in *story* and recovered rapidly already during *post1*, defining therefore what was called a “short-lasting” pattern of changes.

## Discussion

This paper shows that the cerebellum is deeply entrained in well-structured RSN clusters, which reflect multiple aspects of cognitive processing during and beyond the conclusion of auditory stimulation. While it is well known that RSNs (Beckmann et al., 2005; Buzsaki, 2006; Sadaghiani et al., 2010; Sporns, 2013) can change their FC in some physiological and pathological conditions (Hasson et al., 2010; Kauppi et al., 2010), this paper further demonstrates that appropriate paradigms can capture RSN changes occurring when brain activity switches from the actual resting state to a continuous cognitive processing (two states that we will call *quiescent* and *engaged*) during a *naturalistic stimulation*, i.e., while listening to a narrated story.

In this study, 15 RSNs were identified (**Figure 2**), comprising: SMN, LVN, MVN, AN, which are directly implicated in sensory processing; DMN, FCN, ECN, RVAN, LVAN, TPN, PN, SN, LN, WMN, which are associated with higher cognitive functions (Papanicolaou, 2017); and CBLN, which is usually considered a sensorimotor network, but has recently been correlated to cognitive processing too (Stoodley, 2012). In addition to CBLN, 9 out of the 15 RSNs showed clusters in the cerebellum, including both sensory networks (LVN and MVN) and cognitive networks (DMN, ECN, RVAN, LVAN, SN, LN, and WMN). Interestingly, in addition to frontal and occipital cortex, *the posterior cerebellum showed amongst the most marked FC changes* inside these networks. This observation supports the role of the cerebellum in processing movement and mental manipulation, error/novelty detection, working memory and planning through extended cerebro-cerebellar loops (Palesi et al., 2017).

The switching of brain state from *quiescent* to *engaged* and then back to *quiescent* over a time scale of several minutes, was associated with a marked change in RSNs activity (**Figures 3, 7B**). Local FC was changed during the *story* in all the RSNs, consistent with the knowledge that attentive brain activity is associated with BOLD signal changes (Buzsaki, 2006; Huettel et al., 2009; Hasson et al., 2010; Deco et al., 2011; Golkowski et al., 2017). Consistently, several brain areas showed significantly higher FC during the *story* than before or after it. The visual networks (LVN and MVN) and the cognitive networks (TPN, LVAN, SN, and DMN) were those presenting the largest areas of increased FC (**Figures 4, 5**). This is not surprising given that MVN (which involves the primary visual cortex, V1), and DMN are involved in mental imagery (Zvyagintsev et al., 2013; Pearson et al., 2015; Zhang et al., 2018), while DMN and SN are known to play a key role in attention switching (Seeley et al., 2007; Menon, 2011). There are only far and few articles considering changes during naturalistic stimuli. Some papers reported an FC reduction during naturalistic stimulation (Fransson and Marrelec, 2008; Hasson et al., 2009; Arbabshirani et al., 2013), but a direct comparison is difficult since they used acquisition and analysis protocols very different from ours (e.g., seed-based approaches and connectomics). Other studies comparing FC at rest versus speech listening report indeed strengthened connectivity among the language related brain regions during the naturalistic stimulation (Hampson et al., 2002). Moreover, Shirer et al. (2012), using whole brain connectivity, reports increased FC during memory and subtraction tasks among task-related regions compared to rest condition. A strengthening of connectivity may indeed subtend the requirement for high-level functional integration among distant brain areas during cognitive processing.

**FIGURE 8:**
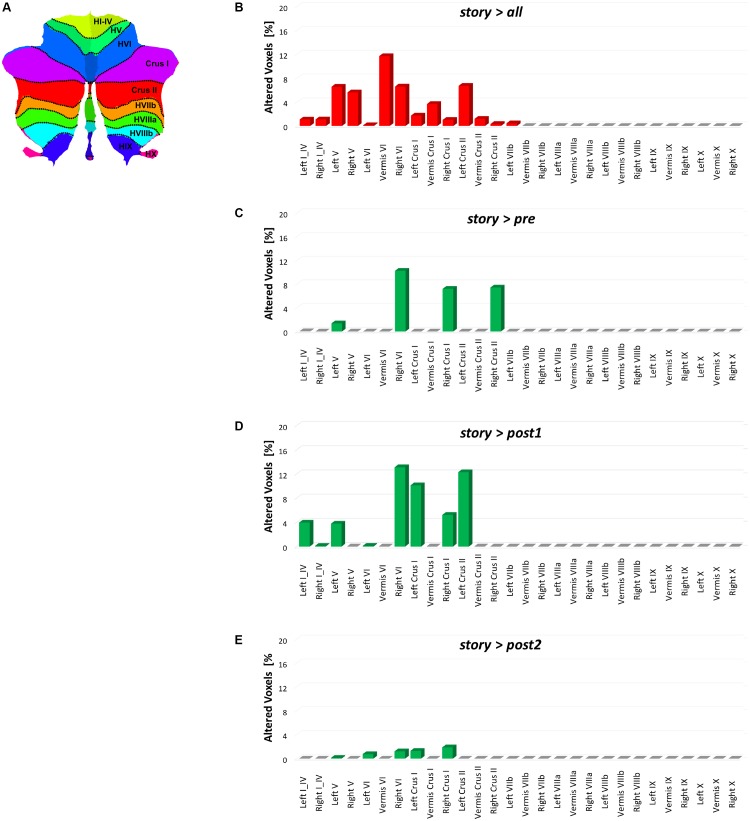
Bar plots showing for each discussed contrast: **(B)** *story* > *all*, **(C)** *story* > *pre*, **(D)** *story* > *post1*, and **(E)** *story* > *post2*, the localization of FC changes within the 28 areas of the cerebellum identified with the SUIT atlas **(A)** (Diedrichsen et al., 2009). For each contrast, each bar height corresponds to the percentage of FC alteration observed in the specific cerebellar area (i.e., number of altered voxels divided for the number of voxels of the area mask in the SUIT atlas). Note that for each contrast, the most altered cerebellar areas are those located in the posterior cerebellum.

This study shows a strong cerebellar involvement in cognitive processing of a narrated story as demonstrated by the characteristic localization of FC changes (**Figure 8**). None of these areas are localized in the anterior cerebellum, which is related to motor control. The cerebellar changes are all localized in the posterior lateral cerebellum, primarily involving Crus-I, Crus-II, and lobule VI, that have previously been related to cognitive processing (Stoodley, 2012). Looking at **Figure 2** it is evident that, these cerebellar areas are part not only of the CBLN, but they are also nodes shared with high-order cognitive networks, such as those processing working memory, attention, internal versus external state switching and language (WMN, VANs, DMN, SN, LN). Specifically, activation in lobule VI could be related to mental rotation or spatial transformations of objects, as observed during fMRI tasks (Vingerhoets et al., 2002; Zacks et al., 2002; Weiss et al., 2009; Stoodley et al., 2010). Activation in Lobule VI and Crus-I could be related to language processing, as demonstrated during reading and lexical decision making tasks (Booth et al., 2007; Carreiras et al., 2007), also in connection with basal ganglia (Booth et al., 2007), and during emotional processing elicited by actions observed in others (Singer et al., 2004; Schulte-Rüther et al., 2007).

The dynamic cognitive processing required for elaborating the story is thought to involve multiple operations (Eysenck, 2012) that can be summarized as follows. Semantic content is analyzed to extract the information required to generate, in association with previous memory, an internal representation of objects and scenes (visual mental imagery). This representation has to account for relative movement and involves mental manipulation (spatial transformations and rotation) of the objects in time and space. Working memory is required to bind temporally distant elements, while error/novelty-detection allows the identification of violations of expectation elaborated on the basis of the ongoing information flux. The occurrence of unexpected elements (either “wrong” or novel) determines attention switching and stimulates planning of new internal schemes. At the same time, some learning processes are expected to take place in order to memorize the story and follow its content. How are our results fitting with this overall processing scheme? The network changes that we observed in our study automatically identified, indeed, three clusters of RSNs that we could speculate are associated with three main mental functions: mental imagery, attention switching and working memory (C1, C2, C3 in **Figure 7A**). Mental imagery can be associated to the cluster (C1) that includes the visual networks MVN and LVN. Both these networks are mainly located in the occipital lobe and include the primary visual cortex (V1) that has been shown to be involved in visual mental imagery (Knauff et al., 2000; Pearson et al., 2015). The C2 cluster including DMN, SN, CBLN, and LVAN can be associated to attention switching, as this action is known to involve SN and DMN (Menon, 2011), as well as to the error/novelty detection task for which the cerebellum, entirely included in CBLN, has been proposed to play a key role (Anderson et al., 2012; Picerni et al., 2013). Finally, FCN, ECN, and WMN, which grouped in cluster C3, can be associated to working memory involvement in the story listening. Indeed, these networks involve primarily the frontal and prefrontal cortices including BA9-10 and BA44-47 areas which are known to be active areas during working memory tasks (Ranganath et al., 2003). Thus, the three main functions that are supposed to involve the cerebellum along with corresponding cerebro-cortical areas during cognitive processing (D‘Angelo and Casali, 2013) are identified in the three RSN clusters.

It is interesting to note that cluster C3, which includes all the RNSs primarily supporting working memory (WMN and FCN) and executive functioning (ECN), showed a *short-lasting* change overall (**Figure 7B**). This finding may be interpreted as a further indication that these three RSNs may be actively engaged in the sensorial perception of the story as well as in the cognitive processing of its content through an immediate increase of attentional processing. Conversely, cluster C2, which includes the attentive network LVAN, CBLN as well as DMN and SN, and is involved in the external-internal switching loop of attention, showed a *long-lasting* RSN change (**Figure 7B**). We speculate that this long-lasting change supports an emotional and attentional involvement related to the story content that persists after the naturalistic stimulation. Although some indication that this might happen a few seconds after the stimulus was reported in Hasson et al. (2009), our results indicate that this persistence of RSNs FC alterations can last for up to 15 min and involve large scale networks including the cerebellum.

Considering the specific cerebellar areas, visual imagery probably involved lobule VI and Crus-I in MVN as well as in LVN, providing the basis for spatial transformations and mental rotation of objects as well as movement perception and planning (Cattaneo et al., 2014; Ferrari et al., 2018). Language processing in LN was associated to FC change in lobule VI, Crus-I and Crus-II. While the role of cerebellum in visual and language processing is rather well established, somehow more surprising were the remarkable changes occurring in the attentive networks LVAN and RVAN involving lobule VI, Crus-I and Crus-II, implying a fundamental role of these cerebellar areas in attention. These findings allow us to speculate that the cerebellum was actively taking part to attention switching in relation to violations of expectations emerging from visual and semantic representations of the story content, as much as reported for sensorimotor control (Brooks et al., 2015). The active role of cerebellum in switching from the internal to external reference framework during the story can be supported when looking at clustering results of the network behaviors where the CBLN is grouped with DMN and SN, known to play a key role in performing this operation (Menon, 2012).

### Methodological Considerations

Since in this study data were acquired using a sequence with relatively long TR (>2 s), the analysis was limited to FC below 0.1 Hz (Damoiseaux et al., 2006). Recent studies have shown that spontaneous BOLD activity may also persist in higher frequency bands (up to 0.8 Hz) (Boubela et al., 2013; Chen and Glover, 2015). It would be interesting to extend our study acquiring rs-fMRI with a multi-band EPI sequence, which would enable the investigation of changes of spontaneous BOLD activity above 0.1 Hz. This would also allow to adopt different analysis approaches, such as sliding window (SW) analysis (Shakil et al., 2016), inter-subject correlation (ISC) analysis (Hasson et al., 2010) and dynamic time warping (DTW) analysis (Meszlényi et al., 2017), that might reveal further time-varying aspects of RSN FC dynamics.

In this study, the naturalistic stimulation was represented by a story enriched with elements that are known to engage working memory and executive functions supported by the cerebro-cerebellar circuits. Here, we aimed to explore overall changes caused by this naturalistic stimulus through the analyses of the RSNs. The study of the contribution of each single element of the story to the FC changes was beyond the purposes of our work as would require a different acquisition strategy for example to allow a dynamical FC analysis. Future studies may be designed to assess individual elements contributions to RSNs FC changes or to assess RSNs FC alterations using different naturalistic stimuli, e.g., visual scenes or less engaging story contents.

## Conclusion

The RSNs FC changes detected during continuous cognitive processing, which involved working memory along with attention switching and object mental manipulation, deeply involved the posterior cerebellum. Beyond demonstrating the existence of structural connections between the cerebellum and several cortical structures, this can be considered as clear evidence of the functional engagement of the cerebellum during cognitive processing. The specific role of cerebellum within these networks can be further analyzed considering brain theories that place the cerebro-cerebellar circuits at the core of processes of error detection and sensory prediction (D‘Angelo and Casali, 2013; Ito, 2013). Similarly, one should construct theories to understand how activity in these circuits is perpetuated beyond the story listening potentially to promote interpretation and memorization. Future studies could optimize recovery timings further in the experimental protocol in order to analyze these novel concepts in greater detail. Given the emerging involvement of the cerebellum in several neurological and neuropsychiatric disorders, one could envisage the use of this simple protocol to assess mechanisms of cognitive alterations in pathologies such as Alzheimer’s disease, Multiple Sclerosis and autism, just to name a few (Castellazzi et al., 2014; d’Ambrosio et al., 2017; Olivito et al., 2018).

## Author Contributions

GC, CW-K, and ED’A conceptualized the study. GC designed and performed the rs-fMRI analysis. SB designed and wrote the narrated story that was used as naturalistic task in the study. GS and FP helped with the setting of the rs-fMRI sequence on the MRI scanner. CW-K and ED’A provided the support and guidance with data interpretation with the contribution of AT, LC, and GS. GC, CW-K and ED’A wrote the manuscript, with comments from all other authors.

## Conflict of Interest Statement

The authors declare that the research was conducted in the absence of any commercial or financial relationships that could be construed as a potential conflict of interest. The reviewer GO and handling Editor declared their shared affiliation.

## Footnotes

1 http://www.fmrib.ox.ac.uk/fsl/
