# Supplementary material for: Prominent Changes in Cerebro-Cerebellar Functional Connectivity During Continuous Cognitive Processing
Source: Front Cell Neurosci. 2018 Oct 1;12:331. doi: 10.3389/fncel.2018.00331 (PMC6174227; doi:10.3389/fncel.2018.00331)
Supplement: Supplementary file 1 [file Image_1.PDF]

Supplementary Material

Prominent changes in cerebro-cerebellar functional connectivity during continuous cognitive processing

G. Castellazzi*, S.D. Bruno, A.T. Toosy, L. Casiraghi, F. Palesi, G. Savini, E. D'Angelo, C.A.M. Gandini Wheeler-Kingshott

*** Correspondence:** Gloria Castellazzi, gloria.castellazzi@unipv.it

1 Supplementary Data

Text of the listening task (i.e. the story) and verification questionnaire

Here we report the text of the story (in Italian and in English language) we used as naturalistic stimulation in our experimental rs-fMRI protocol. In the present study, the story was delivered in Italian to healthy native Italian speaking subjects with comparable education level (all hold a university degree). The English version of the story has been prepared for further exploitations in other studies.

Color-coded elements included to enrich the story to stimulate cerebellar involvement have been highlighted:

Red: *movement and mental manipulation*
Magenta: *error/novelty detection*
Green: *planning*
Blue: *working memory*

Overall, we estimated that the story contains 40 elements (numbered in brackets) specifically included to stimulate cerebellar involvement as described in the manuscript. Here in *italics* is the entire text that was read to the subjects:

1.1 Italian version

SCUOLA DI MAGIA

Peter alzò gli occhi sulla lavagna e rilesse l'elenco degli ingredienti:

- *sangue di drago*
- *dente di pipistrello*

- *ali di falena*
- *bava di rospo*
- *pelo di gatto nero*

La pozione invisibilizzante in effetti non era molto appetitosa. Però valeva la pena impararla bene, per consentirsi qualche scappatella fuori dalle mura. Correva voce che tempo fa un allievo del quinto anno avesse dimenticato un ingrediente e non si fosse accorto di essere diventato invisibile tutto, tranne la testa, che (1) era stata vista ballonzolare sorridente sul sentiero che conduce al paese. Si era beccato un mese di detenzione! Peter tentò di riconcentrarsi sulla pozione. In realtà la parte più difficile era materializzare il calderone davanti a sè: bastava una piccola distrazione perché apparisse al posto sbagliato, magari sulla testa del Prof! Chiuse gli occhi e iniziò a visualizzare un grosso calderone di rame a circa due metri da sè. Eccolo qua. (2) Adesso doveva farlo arrivare sul banco. Si concentrò più intensamente e (3) il calderone iniziò a vibrare, e poi, lentamente, a (4) rotolare su sè stesso (5) avvicinandosi. (6) Atterrò sul bancone di legno con un rumore sordo. Pfeww…nessun incidente! Nel frattempo gli ingredienti della pozione erano scomparsi dalla lavagna: rimanevano visibili solo per un minuto, (7) luccicando in brillanti caratteri verdi, poi si sbiadivano rapidamente fino a dissolversi. Allora, c'era: (8) sangue di drago, dente di pipistrello, ali di falena, bava di rospo e pelo di gatto…? Accidenti, di che colore era sto gatto?! Ci riflettè un attimo. E' risaputo che i gatti con poteri magici più forti sono quelli rossi… (9) aprì il barattolo di vetro e (10) buttò dentro la mistura un ciuffo di (11) peli fulvi. Un odore nauseabondo si levò dal calderone mentre il contenuto evaporava rapidamente. Peter guardò sconsolato il fondo bruciaticcio. Ok, doveva essersi sbagliato. (12) Ora doveva ricominciare dall'inizio: (13) sangue di drago, dente di pipistrello, ali di falena, bava di rospo – (14) questa volta proviamo gatto nero. Nessuna reazione avversa evidente. Peter (15) immerse il dito nella mistura, (16) lo estrasse lentamente e lo guardò (17) scolorirsi fino a scomparire nel giro di qualche secondo. He he, stavolta aveva funzionato. Si chiese se sarebbe riuscito a sparire del tutto senza che il prof se ne accorgesse. Il suo migliore amico James stava facendo il compito in classe di lettura di tarocchi e sicuramente era nei guai. (18) Se Peter avesse potuto diventare invisibile e andarsi a posizionare dietro la sedia di James avrebbe potuto provare a muovere le carte al suo posto. (19) Cercò di visualizzare gli ostacoli che avrebbe incontrato. Un problema era sicuramente quello di uscire dalla porta, dato che le massicce porte di quercia avevano una serratura a tempo che si apriva solo alla fine della lezione. Sarebbe dovuto (20) uscire dalla finestra e (21) camminare sul cornicione in pietra fino al finestrone della stanza accanto, sperando che fosse aperto. Iniziò a preparare una dose maggiore di pozione: (22) sangue di drago, ali di pipistrello… Nel frattempo nell'aula affianco James fissava sconsolato le grosse carte colorate sparse sul tavolo davanti a sè. Giacevano lì, immobili come cadaveri. Dai tavoli dei compagni invece (23) si levavano mazzi di carte fruscianti che si (24) rimescolavano e assortivano nell'aria prima di (25) ricadere sui banchi in nuove misteriose combinazioni da decifrare. Il rumore era assordante, come se centinaia di (26) uccelli impazziti svolazzassero nella stanza. James si era dato malato il giorno della spiegazione e non aveva imparato la formula magica che rendeva le carte animate. Per fortuna aveva con sè nello zaino una bocciona di pozione invisibilizzante che aveva preparato preventivamente il giorno prima. (27) Se fosse riuscito a diventare del tutto invisibile sarebbe potuto sgattaiolare nell'aula vicina a farsi dare qualche suggerimento da Peter. In effetti, le porte in (28) pelle di drago sarebbero rimaste serrate fino alla fine della lezione ma i grandi finestroni erano aperti. Peter guardava impaziente le sue scarpe ancora visibili sul pavimento. Era riuscito con un (29) gesto fulmineo a rovesciarsi il contenuto del calderone in testa mentre il Professore era girato ma la pozione era piuttosto densa e ci aveva messo un po' ad (30) arrivare ai piedi. Ancora qualche secondo di pazienza e (31) avrebbe potuto iniziare a muoversi verso la finestra.

(32) James si mosse cautamente verso il finestrone. Non doveva fare assolutamente alcun rumore che attirasse l'attenzione nella sua direzione. Purtroppo nella foga gli era scappata un po' la mano e aveva fatto scomparire anche mezzo tavolo, con la parte rimanente dubbiosamente in equilibrio su due gambe. Per fortuna nella confusione di (33) tarocchi svolazzanti non sembrava essersene accorto nessuno. (34) Peter raggiunse la finestra e (35) si issò sul davanzale di pietra. (36) James mise un piede sul cornicione. (37) Ancora pochi passi e … BONK! Uno scontro frontale fece perdere l'equilibrio a entrambi e si sentirono (38) spinti nel vuoto da una forza invisibile. Iniziarono a (39) precipitare verso il cortile della scuola. Speriamo che uno dei due riesca a (40) materializzare un materasso sul prato sottostante prima dell'atterraggio!

====================

A questionnaire was also delivered to the subjects at the end of the MRI session, in order to check their understanding and recollection of the story. The score of the questionnaire was added to the dual regression analysis as additional covariate. The mean and standard deviation value of the score across subject was (5.0±0.0) out of a maximum of 5.

Here in italics is the original questionnaire:

Questionario

Scan ID ________________________________

Data ________________________________

1) *Di cosa parla la storia?*
    a. *Una scuola in Inghilterra*
    b. *Una lezione di licantropia*
    c. *Un'avventura spaziale*
    d. *Una scuola di magia*
    e. *Non lo so*
2) *Quanti personaggi ci sono nella storia?*
    a. *Uno*
    b. *Due*
    c. *Più di due*
    d. *Non lo so*
3) *Cosa succeed al calderone?*
    a. *Si rompe*
    b. *Si brucia*
    c. *Si muove nello spazio*
    d. *Impazzisce*
    e. *Non lo so*
4) *Di che colore è il pelo del gatto nella lista degli ingredienti?*
    a. *Nero*
    b. *Rosso*

c. *A macchie*
d. *Non lo so*

5) *Qual è il potere della pozione?*
   a. *Ti rende invisibile*
   b. *Ti restringe*
   c. *Ti fa ingrassare*
   d. *Non lo so* ☐

Ti sei addormentato durante l'esame di risonanza?

____

Se SI, quando?

____

Hai avuto difficoltà a non addormentarti?

____

Se SI, quando?

____

1.2 English version

--

SCHOOL OF MAGIC

Peter looked up at the blackboard and once again read the list of ingredients:

- *dragon's blood;*
- *bat's tooth;*
- *moth's wings;*
- *toad's foam;*
- *hair of a black cat.*

One has to say that the invisibility potion wasn't very appetizing. Still worth trying to master it, though, as it would come in very handy for the odd secret excursion outside the walls. There were rumours that a fifth year pupil forgot an ingredient and didn't realize that he had become entirely invisible, except for his head, (1) which was seen bobbing up and down along the path to the village. One month of detention he got!!

Peter tried to focus on mixing the potion. The most difficult bit was conjuring up the cauldron in exactly the right spot in front of you. Just a tiny distraction and it could land in the wrong place; on the Professor's head, for example! He closed his eyes and started to visualize a large copper cauldron about two metres away. Here it comes. (2) Now he needed to guide it safely to the desk. He concentrated more intensely and (3) the cauldron started to vibrate and then (4) rotate slowly, (5) getting nearer and nearer. (6) At last it landed on the large wooden table with a hollow thud. Phew… no accidents! In the meantime, the list of ingredients had disappeared from the blackboard. They were always only visible for one minute, (7) glittering in bright green letters, before gradually growing fainter and vanishing. So, it was… (8) dragon's blood, bat's tooth, moth's wings, toad's foam and hair of … Damn! What colour was the cat? Peter thought about it for a minute … Mmmh, it's well known that the most powerful cats in magic arts are red cats. (9) He opened the glass jar and (10) threw a tuft of (11) ginger hair into the mixture. A nauseating smell rose up from the cauldron, while the contents quickly evaporated with a hissing sound. Peter stared gloomily at the burnt bottom of the pot. Right…perhaps he had made a mistake. (12) Now he had to start again: (13) dragon's blood, bat's tooth, moth's wings, toad's foam… (14) Let's try black cat this time … no obvious adverse reactions… Peter (15) sank his index finger into the mixture, (16) slowly withdrew it, and watched it (17) discolour and disappear within a few seconds. Hooray, it worked this time! Peter's best friend James was in the middle of his exam in Tarot reading, and was almost certainly in trouble. (18) If Peter managed to disappear, he could place himself behind James's chair and move the cards on his behalf, without anyone knowing. (19) He tried to visualize the obstacles with this plan. A big problem was getting out of the door. The heavy oak doors had a timed lock, which unlocked only at the end of the class. (20) He could climb out of the window though, and (21) walk on the stone ledge to the big window of the adjacent room, hoping it would be open. He started to concoct a larger dose of the potion: (22) dragon's blood, bat's tooth … At the same time, next door, James was staring disconsolately at the large, colourful Tarot cards spread on the table in front of him. They lay motionless, like corpses. Around him, (23) rustling decks of cards shot up from the desks of his classmates, (24) shuffling and reshuffling in mid-air before (25) dropping back on the desks in new mysterious combinations. The noise was deafening, as if hundreds of (26) crazed birds were flapping frantically across the room in all directions. James had feigned sickness on the day of the lecture and had missed out on the instructions on how to animate the cards. Luckily, he had brought along a big bottle of invisibility potion prepared the day before, in case of need. (27) If he managed to make himself invisible he could sneak out to next door and get some help from Peter. The (28) dragon skin doors would stay locked until the end of class, but the big window was open. Peter was staring impatiently at his shoes, still visible on the floor. He had managed to (29) quickly pour the contents of the cauldron on his head while the Professor had his back turned but the potion was rather thick and it had taken a (30) while to reach his feet. A few more seconds of patience, and then (31) he could start to move towards the window. (32) James walked cautiously towards the big windows. He had to be absolutely silent and careful not make any noise that could attract attention. Unfortunately, in his hurry, he had got carried away with the potion and had also made half of his table invisible, the other half now stood suspiciously on two legs. Luckily in the commotion of (33) flying cards nobody seemed to have noticed. (34) Peter got to the window and (35) climbed out onto the ledge. (36) James stood on the ledge. (37) A few more steps and … BONK! An invisible force (38) pushed both off the ledge and they (39) hurtled towards the ground. The schoolyard was getting nearer and nearer … Let's hope that at least one of them (40) manages to conjure up a mattress before landing!

Questionnaire

Scan ID ______________________________

Date ______________________________

1) *What is the story about?*
    a. *A school in England*
    b. *A lycanthropy lesson*
    c. *A space adventure*
    d. *A school of magic*
    e. *I don't know*
2) *How many main characters take part in the story?*
    a. *One*
    b. *Two*
    c. *More than two*
    d. *I don't know*
3) *What happens to the caldron?*
    a. *It breaks*
    b. *It turns on*
    c. *It moves in space*
    d. *It goes mad*
    e. *I don't know*
4) *What colour was the cat's hair in the ingredients list?*
    a. *Black*
    b. *Red*
    c. *Spotty*
    d. *I don't know*
5) *What was the power of the potion?*
    a. *To make you invisible*
    b. *To shrink you*
    c. *To fatten you*
    d. *I don't know*
6) *What was James doing?*
    a. *A maths test in school*
    b. *A tarot test*
    c. *Was listening to a lesson of magic*
    d. *I don't know*

Did you fall asleep during the MRI experiment?

____

If YES, when?

____

Did you find it hard not to fall asleep?

____

If YES, when?

____
